# MicroRNAs targeting Nicastrin regulate Aβ production and are affected by target site polymorphisms

**DOI:** 10.3389/fnmol.2014.00067

**Published:** 2014-07-18

**Authors:** Charlotte Delay, Véronique Dorval, Alice Fok, Benjamin Grenier-Boley, Jean-Charles Lambert, G.-Y. Hsiung, Sébastien S. Hébert

**Affiliations:** ^1^Axe Neurosciences, Centre de Recherche du CHU de Québec, Université Laval, QuébecQC, Canada; ^2^Département de Psychiatrie et de Neurosciences, Faculté de Médecine, Université Laval, QuébecQC, Canada; ^3^Institut Pasteur de Lille, INSERM U744, Université Lille Nord de FranceLille (Nord), France; ^4^Division of Neurology, Department of Medicine, University of British ColumbiaVancouver, QC, Canada

**Keywords:** Alzheimer’s disease, Nicastrin, microRNA, single-nucleotide polymorphism, miR-186, miR-455

## Abstract

Despite the growing number of genome-wide association studies, the involvement of polymorphisms in microRNA target sites (polymiRTS) in Alzheimer’s disease (AD) remains poorly investigated. Recently, we have shown that AD-associated single-nucleotide polymorphisms (SNPs) present in the 3′ untranslated region (3′UTR) of amyloid precursor protein (APP) could directly affect miRNA function. In theory, loss of microRNA (miRNA) function could lead to risk for AD by increasing APP expression and Aβ peptide production. In this study, we tested the hypothesis that Nicastrin, a γ-secretase subunit involved in Aβ generation, could be regulated by miRNAs, and consequently affected by 3′UTR polymorphisms. Bioinformatic analysis identified 22 putative miRNA binding sites located in or near Nicastrin 3′UTR polymorphisms. From these miRNA candidates, six were previously shown to be expressed in human brain. We identified miR-24, miR-186, and miR-455 as regulators of Nicastrin expression, both *in vitro* and under physiological conditions in human cells, which resulted in altered Aβ secretion. Using luciferase-based assays, we further demonstrated that rs113810300 and rs141849450 SNPs affected miRNA-mediated repression of Nicastrin. Notably, rs141849450 completely abolished the miR-455-mediated repression of Nicastrin. Finally, the rs141849450 variant was identified in 1 out of 511 AD cases but not in 631 controls. These observations set the stage for future studies exploring the role of miRNAs and 3′UTR polymorphisms in AD.

## INTRODUCTION

The small, non-coding miRNAs function as important regulators of protein expression (Ambros, 2004). They act by imperfect base-pairing to target messenger RNAs, leading to translational repression, degradation, or both (Pillai, 2005). Important for this action is the seed sequence, located at positions 2–8 within the miRNA sequence. It is now well documented that specific miRNAs are altered in a number of neurodegenerative disorders including Alzheimer’s disease (AD; reviewed in Delay et al., 2012). These observations evoke the hypothesis that miRNA dysregulation could directly be implicated in the disease processes, either detrimental or as an attempt of neurons to survive. Moreover, increasing evidence suggests that single-nucleotide polymorphisms (SNP) within miRNA target sites might contribute to disease risk. For instance, the Parkinson’s disease-associated rs12720208 SNP is located in the miR-433 target site of Fibroblast growth factor 20, and affects miRNA-mediated repression (Wang et al., 2008). The rs5848 SNP, associated with TDP43-positive frontotemporal dementias, is located in the miR-659 target site of the Progranulin gene, also affecting the miRNA-mediated repression (Rademakers et al., 2008). Previously, we have shown that AD-associated SNPs present in the untranslated region (3′UTR) of APP could directly affect miRNA function (Delay et al., 2011). In theory, loss of miRNA function could contribute significantly to risk for AD by increasing APP expression and therefore Aβ production. Aβ peptides are the major constituents of amyloid (senile) plaques that accumulate in AD brain (Glenner and Wong, 1984).

Another essential player in Aβ production is the γ-secretase complex, composed of four subunits (i.e., Presenilin, Nicastrin, Aph-1, and Pen2; De Strooper, 2003). Recent studies have documented rare Nicastrin (NCSTN) variants associated with AD (Lupton et al., 2011), whereas studies in mice have shown that increased expression of NCSTN is sufficient to promote AD pathology (Goo et al., 2013). In this study, we evaluated whether miRNAs could modulate NCSTN expression, and whether polymiRTS within the 3′UTR of the Nicastrin gene could abrogate miRNA function.

## MATERIALS AND METHODS

### PATIENTS

Cases of AD and controls were participants from two large Canadian cohorts: the Canadian Study of Health and Aging (CSHA) and A Canadian Collaborative Cohort of Cognitive Impairment and Related Dementia (ACCORD). Ethical approval of these studies was obtained from the ethics review board in each of the study centers. Details of each cohort have been published elsewhere (Anonymous, 1994; Feldman et al., 2003; Hsiung et al., 2004). The clinical examination was developed in collaboration with the US Consortium to Establish a Registry for Alzheimer’s Disease. AD cases were diagnosed by NINCDS – ADRDA clinical criteria (McKhann et al., 1984), and controls were subjects who remained not cognitively impaired until the end of the study or censorship.

### CELL CULTURE

Human HeLa and HEK293-APPSw cells were cultured in DMEM medium (Invitrogen, Carlsbad, CA, USA) supplemented with 10% heat-inactivated fetal bovine serum. One day before transfection, HeLa cells were plated at 20% confluence in six-well or 24-well plates and 250,000 HEK-APPSw cells were plated into six-well plates. Transfection was performed using Lipofectamine 2000 (Invitrogen, Carlsbad, CA, USA) according to the manufacturer’s instructions.

### cDNA CONSTRUCTS

The full-length *hNCSTN* 3′UTR was extracted by PCR from SH-SY5Y cells using the following primers: forward: 5′-TTTTTCTAGAGGAGGACCCCAGCTTTTC, reverse 5′-AAAAGGATCCCGTGTGGGATAATCTATTTT, and were cloned using XbaI (New England Biolabs) and BamHI (New England Biolabs) enzymes into the pGL3-promotor vector (Promega, USA). Mutagenesis was performed by TOPgene technologies (Montreal, Quebec, Canada) and validated by sequencing.

### LUCIFERASE ASSAY AND PROTEIN ANALYSIS

HeLa cells were transfected with 25 or 50 nM pre-miRs (Applied Biosystems, USA), 2.5 ng/cm^2^; pRL control vector, and 50 ng/cm^2^; pGL3_HSV TK_3′UTR hNCSTN WT or C460T (*rs1043329*), T623G (*rs113810300*) or delCA515–516 (*rs141849450*) mutant variant plasmids. Twenty-four hours post-transfection, cells were lyzed, and luciferase activity was measured according to the manufacturer’s instructions (Promega, USA). For western blots, cells were lyzed in RIPA buffer [50 mM Tris HCl, 1% NP40, 0.9% NaCl, 0.25% Na-deoxycholate, 1 mM EDTA, 1× proteinase inhibitors (Roche, Basel, Switzerland), 1 mM PMSF, 1 mM Na_3_VO_4_, and 1 mM NaF], mixed with LDS sample buffer (Invitrogen, Carlsbad, CA, USA) containing 5% β-mercapto-ethanol and boiled at 95°C for 8 min. Crude extracts (10 μg) were immunoblotted with the Aph-1a [clone B80.2, kind gift from B. De Strooper (Nyabi et al., 2003)], Pen2 (Cell Signaling, clone D2G6), Nicastrin (Cell Signaling, clone D38F9), Presenilin 1 (Millipore, clone PS1-loop), and Gapdh (Millipore, clone 6c5) antibodies. Membranes were detected using the ECL detection kit (Millipore, Billerica, MA, USA). Quantifications were performed using the Multi Gauge software (FUJIFILM, Minato-ku, Tokyo, Japan).

### ELISA

HEK293-APPSw cells were transfected with 50 nM pre-miRs (Applied Biosystems, USA). Twenty-four hours post-transfection, cell lysates were collected, spun at 1000 × *g* for 10 min to remove debris, and supernatants were kept on ice. Human (soluble) Aβ1–40 and Aβ1–42 peptides were measured using Aβ40 and Aβ42 Human ELISA Kits, following the manufacturer’s conditions (Life Technologies, Carlsbad, CA, USA).

### STATISTICS

Statistical significance of western blots and luminescence quantifications was determined using one-way ANOVA, two-way ANOVA, or Student’s paired *t*-test as indicated in the text. Calculations were made using the GraphPad Prism 5 software.

### GENOTYPING

DNA was extracted from peripheral blood (AutogenFlex STAR, Holliston MA, USA), and SNP genotyping was performed with TaqMan assays in an optimized ABI 7300 (Applied Biosystems). The two SNPs chosen in this study were rs113810300 and rs141849450 in human Nicastrin.

## RESULTS

We first used the National Center for Biotechnology Information (NCBI) database to generate a list of all known SNPs located within the 3′UTR of human NCSTN (**Table 1**). In order to identify SNPs that could affect miRNA binding, we used the on-line algorithms TargetScan and microRNA.org (Betel et al., 2008; Garcia et al., 2011). We included both canonical (i.e., perfect seed match) and non-canonical (i.e., containing G:U wobbles or seed mismatches) miRNA binding sites. This analysis resulted in a list of 22 miRNAs (**Table 1**), which we narrowed down to six (i.e., miR-24, miR-186, miR-340, miR-455, miR-656, and miR-1301) based on our previous expression profiling studies in the human cerebral neocortex (45 raw reads cut-off; Hébert et al., 2013). Whether excluded miRNAs are expressed at higher frequency in other brain regions and/or tissues remains to be determined.

**Table 1:** Polymorphisms located in or near miRNA target sites located in the 3′UTR of *hNCSTN.*

SNPID	Position in 3′UTR	Polymorphism	Predicted microRNA	Seed region	Number of raw reads
Ts10059	18	C/T	hsa-miR-31	Y	0
Ts41266889	196	C/T	hsa-miR-3153	Y	0
Ts180769907	360	A/T	hsa-miR-1226*	Y	0
			hsa-miR-608	N	0
			hsa-miR-92a*	N	0
Ts1043230	367	C/A	hsa-miR-92a-2*	Y	0
			hsa-miR-4298	N	0
Ts1043329	460	C/T	hsa-miR-24	N	150
Ts141849450	515–516	delCA	hsa-miR-455-5p	Y	95
Ts34629439	582	delT	hsa-miR-1301	N	45
			hsa-miR-590-5p	N	11
			hsa-miR-27b*	N	23
			hsa-miR-582-5p	N	15
			hsa-miR-656	N	107
Ts113810300	623	T/G	hsa-miR-1252	Y	0
			hsa-miR-3125	Y	0
			hsa-miR-340	Y	1708
			hsa-miR-142-5p	Y	20
			hsa-miR-186	Y	1209
			hsa-miR-3121	N	0
			hsa-miR-4311	N	0
Ts71719087	638/639	delAT	hsa-miR-3145	Y	0

*The SNP ID and the nature of the polymorphism are indicated. miRNAs which are predicted to interact with the 3′UTR of hNCSTN are listed, and it is noted when the SNP is located within the seed region of the miRNA in question. Also the number of raw reads (from 1 μg of total RNA) of respective miRNAs in the human cerebral neocortex is noted (see Anonymous, 1994 for details). Y, yes; N, no.*

**FIGURE 1:**
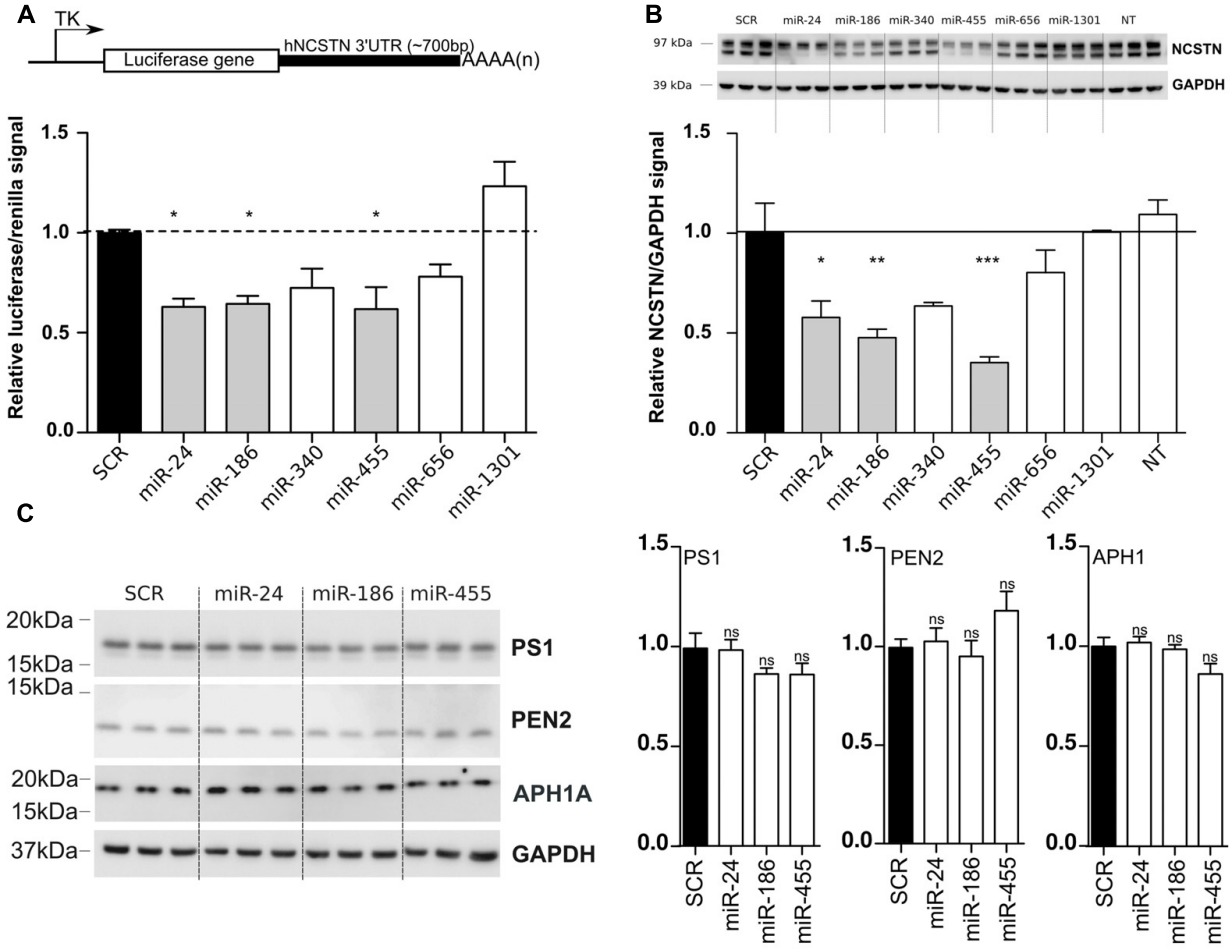
***In vitro* functional analysis of NCSTN targeting miRNAs. (A)** Schematic representation (not to scale) of the luciferase reporter construct used in this study (upper panel). TK; thymidine kinase promoter, AAAA(n); PolyA site, HeLa cells were transfected with 50 nM pre-miRs (as indicated) as well as a reporter construct containing the untranslated region (3′UTR) of *hNCSTN*. The cells were lyzed 24 h post-transfection and luciferase signal was measured. Signals were normalization for transfection efficiency and graph represents the luciferase signals compared to the scrambled control (SCR). Statistical significance was assessed by one-way ANOVA with Bonferroni *post-hoc* test. *p < 0.05. **(B)** HeLa cells were transfected with 50 nM pre-miRNAs (as indicated). The cells were lyzed 48 h post-transfection and western blotting was performed. Representative (*n* = 3, in triplicate) western blots are shown. The ratios of the NCSTN/GAPDH signals are presented (both mature and immature NSCTN). Measurements were normalized to the scrambled control (SCR). Statistical significance was assessed by one-way ANOVA with Bonferroni *post-hoc* test. *p < 0.05, **p < 0.01, ***p < 0.001. **(C)** HeLa cells were transfected with 50 nM pre-miRNAs (as indicated). The cells were lyzed 48 h post-transfection and western blotting was performed. Representative (*n* = 3, in triplicate) western blots and quantifications are shown. Statistical significance was assessed by one-way ANOVA with Bonferroni *post-hoc* test. ns, not significant.

To validate our bioinformatic analyses, we cloned the full-length (∼700 bp) 3′UTR of human *NCSTN* into a luciferase reporter vector (**Figure 1A**), and co-transfected this construct with candidate miRNAs into human HeLa cells. We observed a significant reduction of luciferase expression upon miR-24, miR-186, and miR-455 expression (**Figure 1A**) compared to a scrambled miRNA (SCR) negative control. We also confirmed the downregulation of endogenous NCSTN after transfection of candidate miRNAs into HeLa cells (**Figure 1B**). Notably, only miR-186 and miR-455 decreased both the mature and immature forms of NCSTN (Edbauer et al., 2002). While both miR-340 and miR-656 also affected luciferase and endogenous NCSTN expression, this observation did not reach significance. Overexpression of identified NCSTN targeting miRNAs did not significantly affect endogenous Presenilin 1 (PS1), APH1A, or PEN2 expression (**Figure 1C**). We thus identified miR-24, miR-186, and miR-455 as endogenous regulators of human NCSTN.

**FIGURE 2:**
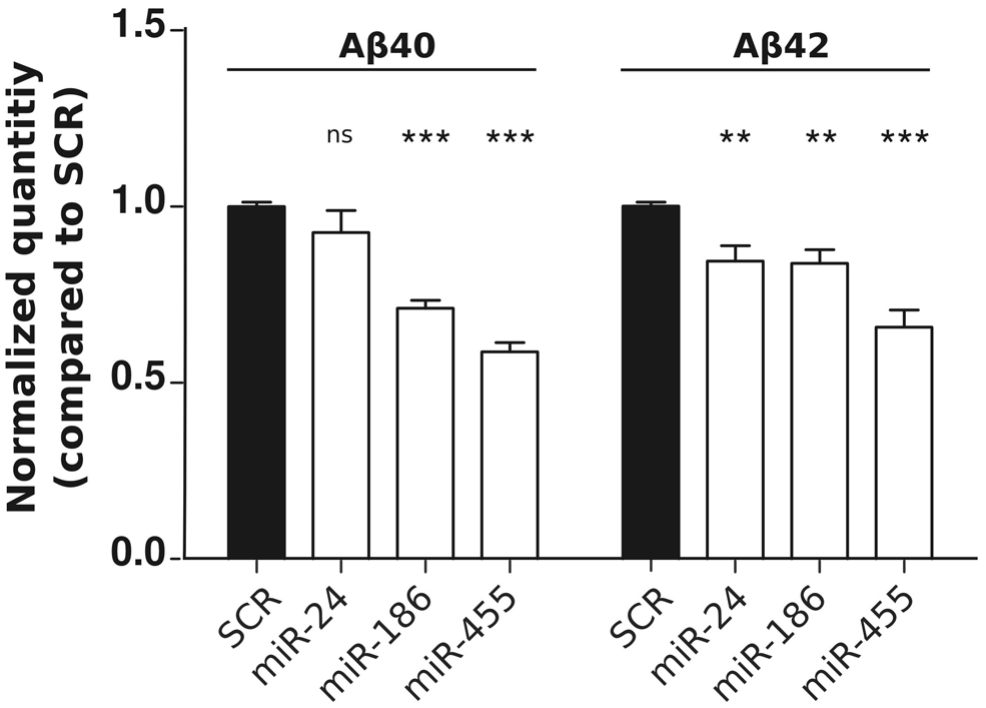
**miR-24, miR-186, and miR-455 expression results in decreased Aβ secretion.** Relative quantities of human Aβ40 and Aβ42 peptides measured by ELISA in the culture medium of HEK-APPSwe cells after transfection with 50 nM pre-miRNAs (as indicated). Signals were normalized to the basal Aβ secretion found in cells transfected with SCR miRNAs. Statistical significance was assessed by one-way ANOVA with Bonferroni *post-hoc* test. *p < 0.05, **p < 0.01, ***p < 0.001.

In order to determine the functional consequences of miR-24, miR-186, and miR-455 expression on Aβ production, we performed ELISA using HEK293-APPSwe cells. Both miR-186 and miR-455 decreased (soluble) Aβ40 and Aβ42 levels, while miR-24 had a small, nonetheless significant effect on Aβ42 (**Figure 2**). The observations correlate with our western blot data (**Figure 1B**), showing that highest NCSTN repression leads to the lowest Aβ secretion.

**FIGURE 3:**
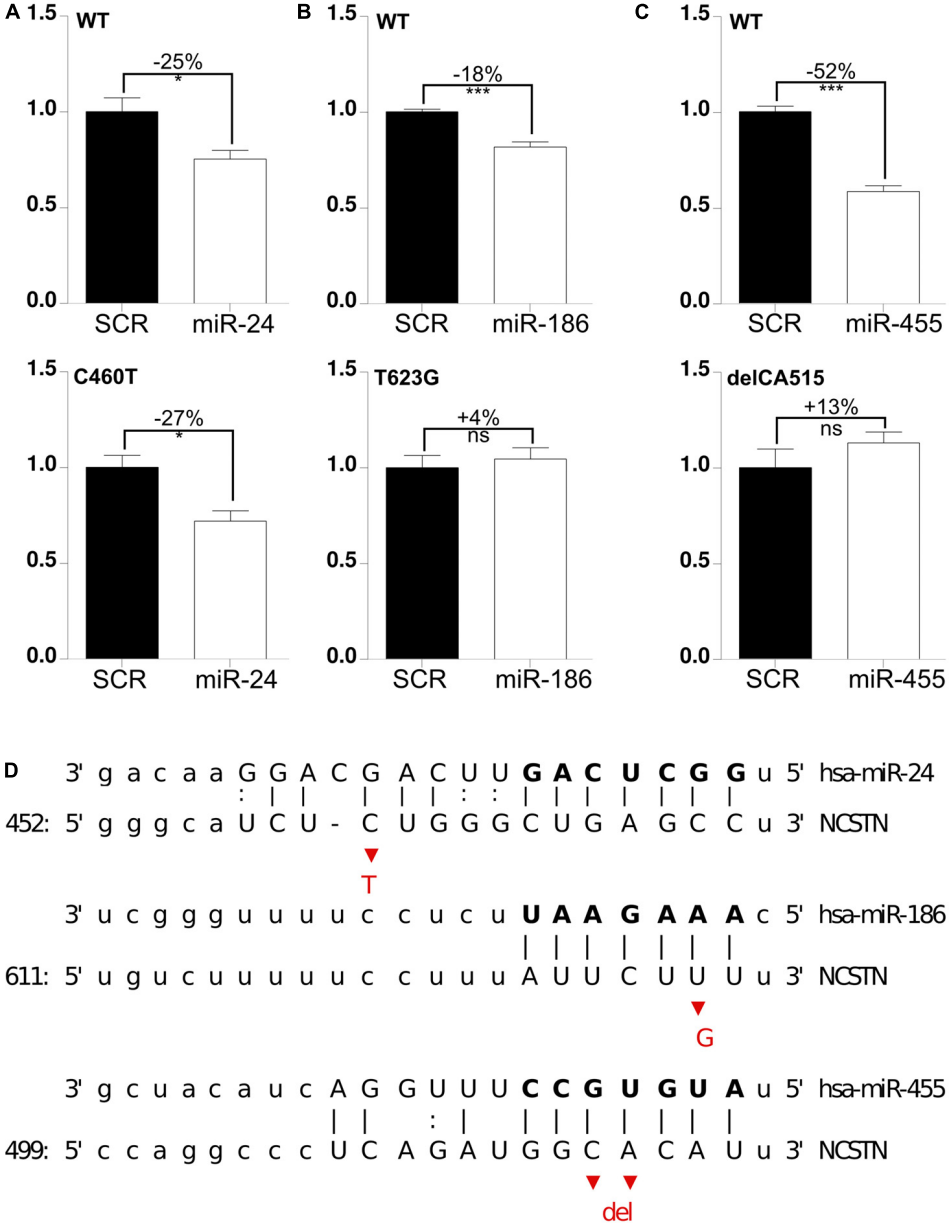
**SNP delCA 515–516 and SNP T623G reduce miR-455- and miR-186-mediated NCSTN repression. (A–C)** HeLa cells were transfected with 25 nM pre-miRs (as indicated) as well as a reporter construct containing the WT, delCA 515–516, T623G and C460T mutated 3′UTR of *hNCSTN*. The cells were lyzed 24 h post-transfection and luciferase signal was measured. After normalization for transfection efficiency, the signals were compared to the SCR control. Representative results (*n*= 3, performed in triplicate) are shown. Statistical significance was assessed by Student’s paired *t*-test (*p < 0.05, ***p < 0.001, ns, not significant). SCR; scrambled. **(D)** Schematic representation of base pair matching between miRNAs and the 3′UTR of *hNCSTN*. The seed region of the miRNAs is indicated. The red bases represent the SNPs C460T (upper panel), T623G (middle panel), and delCA 515–516 (lower panel).

Next, we tested whether these miRNAs were functionally affected by 3′UTR polymorphisms. To address this, we generated human NCSTN 3′UTR luciferase constructs containing the C460T (*rs1043329*), T623G (*rs113810300*), or delCA515–516 (*rs141849450*) variants. As before, these mutant vectors were co-transfected with candidate miRNAs and luciferase activity was measured. These screens indicated that the polymorphism C460T, which is located in the 5′ compensatory region of the miRNA binding site, did not significantly affect miR-24 function (**Figure 3A**). On the other hand, both seed region SNPs T623G and delCA515–516 reduced miR-186 and miR-455-mediated repression, respectively (**Figures 3B–D**). It is interesting to note that both miR-186 and miR-455 have previously been shown to have an altered expression level in AD cerebrospinal fluid (CSF) samples, which further underscores the possible association between these miRNAs and AD development. Taken together, we identified miR-186 and miR-455, which are functionally affected by polymorphisms.

We finally asked whether identified SNPs could be associated with AD. According to the NCBI dbSNP database (February 2014), the rs113810300 SNP has no reported frequency, while the rs141849450 SNP has a 1/1000 frequency in the European reference panel (1000 Genomes Phase I V3 SHAPEIT2, December 2013). Using imputation from this latter panel, rs141849450 has an estimated frequency of 5.6/1000 in an independent French cohort of more than 2000 individuals (data not shown). To validate and extend these observations, we performed TaqMan SNP genotyping assays in a Canadian patient population of AD cases and non-demented controls. These screens identified 1 out of 511 AD individuals harboring the *rs141849450* deletion mutation. This individual was aged 65 years and was a carrier of the APOE4 (e3/e4) allele. Of notice, the *rs141849450* SNP was not present in 631 control. No *rs113810300* polymorphism was identified in either groups, as everyone was homozygous T/T. Given the low frequencies of these SNPs in the general population, much larger cohorts are required to determine whether candidate polymorphisms are indeed associated with disease. Nevertheless, we identified at least one SNP co-segregating with an AD patient, which suggests that rare polymiRTS could contribute to risk for disease in a subset of patients. In theory, the rs141849450 SNP would lead to increased NCSTN expression and Aβ load.

## DISCUSSION

In the current study, we identified a number of brain-expressed miRNAs that could regulate endogenous NCSTN expression and Aβ peptide production. Furthermore, we identified rare 3′UTR polymorphisms that could affect miRNA repressional activity toward NCSTN, at least in our experimental settings. It has been demonstrated recently that, besides expression variation in miRNAs and genetic variants in miRNA loci, polymiRTS (including both SNPs and small insertions and deletions) are important sources of expression variation of miRNA target genes in humans (Gamazon et al., 2012). Moreover, several SNPs that are in linkage disequilibrium with those that might affect miRNA binding have been identified and are associated with the risk of developing disorders including breast cancer, asthma, and Parkinson’s disease (Richardson et al., 2011; Thomas et al., 2011). These observations provide significant evidence for the role of genetic-variation-mediated regulation by miRNAs in the etiology of a broad spectrum of human complex phenotypes, including neurodegenerative diseases, metabolic traits, and autoimmune disorders. However, whether the SNPs identified in this study could also confer risk for AD remains an interesting possibility but will require extensive follow-up studies in larger cohorts of patients. Nevertheless, these observations strengthen the potential role of polymiRTS in neurodegenerative disorders such as AD.

There are some caveats associated with our study. For instance, the luciferase reporter system, although a robust, quantitative method of analysis, has limited clinical relevance. Indeed, the miRNome of immortalized cell lines and a human brain cell are definitely different, making it difficult to account for compensatory or cumulative effects by other miRNAs on the same miRNA target site. Secondly, the basal levels of the miRNA of interest as well as the epigenetics of the region at study might be different between brain and cell lines, possibly resulting in different responses. Validation *in vivo* would be more accurate but would require extensive screening (gene sequencing) studies in patients with AD. Unfortunately, our Canadian patient samples were limited to DNA, so we could not perform *in vivo* Nicastrin expression studies on mRNA. Another alternative would be to generate multiple knock-in cell lines or animals (e.g., mice) harboring candidate polymorphisms within the endogenous NCSTN 3′UTR, which extends the current scope of this study. It should be noticed that current mouse models expressing human NCSTN do not contain the (full) 3′UTR. Moreover, it remains uncertain if the identified miRNAs, although expressed in human brain, are similarly expressed in the mouse brain. Nevertheless, we do provide functional data in human cells which is in line with our main hypothesis that specific miRNAs can regulate Aβ production.

What are the clinical implications of these findings? As mentioned above, this is a notoriously difficult question to answer given the low frequencies of identified SNPs (e.g., 5/1000 for rs141849450) in the general population. These results are somewhat expected and consistent with the notion that functional 3′UTR polymorphisms are uncommon, particularly in the seed region (Chen and Rajewsky, 2006; Saunders et al., 2007; Yu et al., 2007), likely because of evolutionary pressure. They are nevertheless important for human disease, as polymiRTS have been associated with a wide range of diseases, including neurodegenerative disorders such as Parkinson’s disease (Wang et al., 2008). Thus, larger cohorts are required to determine whether candidate polymorphisms might be associated with AD. Of notice, we also explored the frequency and possible correlation between Aβ load and the rs141849450 Nicastrin variant in a large French (European) cohort. Unfortunately, we could not conclude anything due to the lack of SNP-bearing subjects (unpublished observations). We do, however, provide experimental validation that the rs141849450 Nicastrin variant exists in at least one AD patient (Canadian cohort). This is important since not based only on prediction algorithms and imputation values. Whether this has clinical relevance is interesting, but it requires extensive follow-up studies in much larger cohorts of patients.

In conclusion, we identified NCSTN-targeting miRNAs (miR-24, miR-186, and miR-455) that could decrease Aβ secretion. MiR-186 and miR-455 were previously shown to be altered in AD CSF samples, and we demonstrated that the miRNA-mediated repression of NSCTN mRNA is altered by the presence of SNPs rs113810300 and rs141849450. Our data provide additional proof of principle that polymiRTS could contribute to neurodegenerative disorders, and that miR-186 and miR-455 could be important players in AD pathology. We also stress the importance of including non-translated regions in genome-wide association studies.

## AUTHOR CONTRIBUTIONS

Charlotte Delay, G.-Y. Hsiung, Sébastien S. Hébert: design of the study. Charlotte Delay, Véronique Dorval, Alice Fok: experiments. Benjamin Grenier-Boley, Jean-Charles Lambert, Charlotte Delay: statistical analyses. Charlotte Delay, Sébastien S. Hébert: manuscript writing. All authors read and approved the final manuscript.

## Conflict of Interest Statement

The authors declare that the research was conducted in the absence of any commercial or financial relationships that could be construed as a potential conflict of interest.
